# A retrospective study on findings of canine hip dysplasia screening in Kenya

**DOI:** 10.14202/vetworld.2015.1326-1330

**Published:** 2015-11-22

**Authors:** Peter Kimeli, Susan W. Mbugua, Roger M. Cap, Gilbert Kirui, Tequiero O. Abuom, Willy E. Mwangi, Ambrose N. Kipyegon, John D. Mande

**Affiliations:** 1 Department of Clinical Studies, Faculty of Veterinary Medicine, University of Nairobi, P.O. Box 29053-00625, Kangemi, Kenya; 2 Sercombe Veterinary Surgeons, P.O Box 24878-00502, Nairobi, Kenya

**Keywords:** dogs, hip score, hip dysplasia, Kenya

## Abstract

**Aim:**

The current study was undertaken to evaluate the findings of canine hip dysplasia screening in Kenya.

**Materials and Methods:**

Records for 591 dogs were included in this study. The data was obtained from the national screening office, Kenya Veterinary Board, for the period between the years 1998 and 2014. Monthly screening records were assessed and information relating to year of evaluation, breed, sex, age, and hip score captured. Descriptive statistics of hip scores was computed based on year, sex, age, and breed.

**Results:**

A total of 591 records from the year 1998 to 2014 were retrieved at the National Screening Centre, the Kenya Veterinary Board. Each record was examined and data pertaining to year of screening, the breed, sex, age of the dogs, and the total hip score were recorded. The highest number of dogs screened for hip dysplasia (HD) was in the year 2009 and the lowest in the year 1998. More females than males were screened for HD and the mean age of all the dogs was 22.9±12.7 months. The most common breeds of dogs screened during the study period were German Shepherd (67.0%), Rottweiler (15.6%), and Labrador Retriever (12.2%). The mean hip score for the 591 dogs was 15.1±10.9 and the median 12.0. The mean hip scores per breed were; German Shepherd (16.3±12.1); Golden Retriever (16.0); Hungarian Vizla (15.0); Labrador Retriever (3.0±6.7); Great Dane (13.3±3.2); Rottweiler (12.2±8.2); Doberman (10.3±4.2); Rhodesian Ridgeback (9.6±3.8); and Boxer (9.3±0.6). Based on the hip score, moderate to severe HD was diagnosed in 16.6% of the dogs, mild HD in 32.7%, Borderline HD in 37.7%, fair HD in 6.9%, and good HD in 6.1%.

**Conclusion:**

Canine HD is a common occurrence in Kenya with most dogs suffering mild to border line HD. In addition, German Shepherd and Golden Retriever appear to be the most affected breeds. It is therefore recommended that stringent measures be imposed to dog breeding programs to avoid transmission of this undesirable trait and consequently improve the welfare and the quality of dog breeds in Kenya.

## Introduction

Hip dysplasia (HD) is a common and potentially debilitating orthopedic disease in dogs [1] characterized by laxity of the coxofemoral joint leading to secondary osteoarthritis, pain, and reduction in joint function [2]. The condition is manifested by decreased congruence between femoral head and acetabulum resulting in degenerative damage to the joint [3,4]. Although the etiology of this condition is not fully understood, environmental influences such as obesity, injury at a young age, overexertion on hip joint or round ligament tear at a young age, repetitive motion on forming coxofemoral joint, and excess dietary calcium/vitamin D are said to play key roles. HD primarily affects medium-sized and large-breed of dogs and has high heritability of up to 95% [5-8].

Radiographic techniques have been developed and are widely used to identify dogs which are less affected by the disease for purposes of breeding [9]. Internationally, three scoring modes are widely in use: The Fédération Cynologique Internationale, the Orthopedic Foundation for Animals (OFA), and the British Veterinary Association/The Kennel Club (BVA/KC) [6].

The Kenya Veterinary Board (KVB) is the officially mandated institution that conducts canine HD screening in Kenya on behalf of the East Africa KC (EAKC). This institution has been screening dogs for HD using the BVA/KC system and advocating for breeding of dogs with good hips since 1998 (Personal communication).

The BVA/KC system scores hip joints based on the severity of changes of nine specific morphological radiographic criteria which include Norberg angle; subluxation; cranial acetabular edge; dorsal acetabular edge; cranial effective acetabular rim; acetabular fossa; caudal acetabular edge; femoral head and neck exostoses, and femoral head recontouring [2].

Information on canine hip score findings has not been documented in Kenya. This study was therefore conducted to determine the occurrence of HD and the prevailing scores among canines presented to KVB for hip scoring in the period between 1998 and 2014.

## Materials and Methods

### Ethical approval

This was a retrospective study, and no animal was used. The information was recovered from routinely kept records in the national canine hip screening office, with their permission.

### Source of data

The study included all hip-dysplasia screening records for 591 dogs of different breeds from various breeders across the country. The data was obtained from the national screening office, KVB, for the period between the years 1998 and 2014.

### HD screening

Routinely, all the official hip radiographs are judged by a minimum of two trained and experienced veterinary surgeons appointed by the KVB. The panelists are drawn from both the university and private veterinary hospitals. As a requirement, all the radiographs must bear the dogs’ microchip or tattoo number, which is the registration number of the EAKC, and this is cross-checked with the details on the application form. In addition, radiographs must satisfy the set criteria for accurate scoring and interpretation of hips in dogs.

### Data management

Using monthly screening records, all relevant data that included year of evaluation, breed, sex, age, and hip score was captured. The data from the archived records was entered and stored in Microsoft office excel 2007 (Microsoft Corporation, 2007) and exported to statistical analytical system (SAS) 2002-2003 (SAS Institute, Inc., Cary, NC, USA) for analysis. Descriptive statistics of hip scores was computed based on year, sex, age, and breed. Grading of hip scores was done based on BVA/KC as described by Flückiger *et al*. [6].

## Results

### General demographics

A total of 591 hip score records were examined in this study. Generally, the number of dogs screened for HD increased exponentially from 1998 through to 2014 as shown in Table-1. A similar trend was also observed in the breeds of dogs.

**Table-1:** Breed of dogs with their frequencies and percentage over the years in a retrospective study on the canine HD scheme in Kenya.

Breed	Year																	Total (%)

	1998	1999	2000	2001	2002	2003	2004	2005	2006	2007	2008	2009	2010	2011	2012	2013	2014	
Boxer	-	-	-	-	-	-	-	-	-	1	-	1	-	-	1	-	-	3 (0.51)
Doberman	-	1	-	-	1	-	-	1	1	-	-	2	1	-	-	-	2	9 (1.52)
Great Dane	-	-	-	-	-	-	-	2	1	-	-	-	-	-	-	-	-	3 (0.51)
Golden Retriever	-	1	-	-	-	-	-	-	-	-	-	-	-	-	-	-	-	1 (0.17)
German Shepherd	7	7	13	33	15	8	23	38	32	38	21	34	26	32	30	12	27	396 (67.01)
Hungarian Vizla	-	-	-	-	-	-	-	-	-	1	-	-	-	-	-	-	-	1 (0.17)
Labrador Retriever	-	1	-	-	1	-	-	7	2	9	3	3	5	6	13	7	15	72 (12.18)
Rottweiler	-	3	1	-	1	-	1	6	6	7	6	17	9	10	10	5	10	92 (15.57)
Rhodesian Ridgeback	-	-	-	-	-	-	-	2	5	-	-	3	-	1	3	-	-	14 (2.37)
Total	7	13	14	33	18	8	24	56	47	56	30	60	41	49	57	24	54	591

HD=Hip dysplasia

More female (67.5%) than male (32.5%) dogs were screened for HD. This finding was consistent for all the years of study.

German Shepherds (67.0%) were the most common breed screened for HD during the study period followed by Rottweilers (15.6%) and Labrador Retrievers (12.2%). The least common breeds were Golden Retrievers (0.2%) and Hungarian Vizlas (0.2%).

Dogs screened for HD were aged between 12 and 30 months (mean 22.9±12.7; median 19 months). The greatest number of screened dogs was in the age range of 12-24 months (70.7%; 418/591), followed by 25-48 months (24.2%; 143/591) and more than 48 months (5.1%; 30/591).

### Hip scores

**Figure-1:**
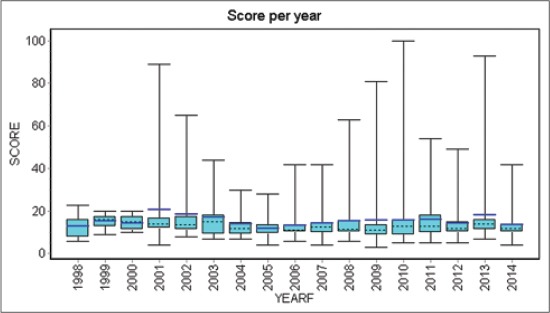
Plot showing the trend of mean hip score over the years.

The mean hip score for the 591 dogs screened during the study period was 15.1±10.9 (median 12.0). The mean hip score for female dogs (16.2±11.6; median 13.0) was higher than that of males (12.8±8.9; median 11.0). Highest mean hip score was in German Shepherds (Mean 16.3±12.1; Median 13.0) whereas the lowest mean score was observed in Boxer (Mean 9.3±0.6; Median 9.0) as shown in Table-2. The mean hip score remained fairly constant over the years (Figure-1).

**Table-2:** HD score descriptions in different dog breeds and sexes in a retrospective study on the canine hip dysplasia scheme in Kenya.

Breed	Sex	Mean	Median	Standard deviation
Boxer	F	9.0	9.0	0.0
	M	10.0	10.0	-
	All	9.3	9.0	0.6
Doberman	F	10.6	10.0	4.8
	M	9.5	9.5	2.1
	All	10.3	10.0	4.2
Great Dane	F	17.0	17.0	-
	M	11.5	11.5	0.7
	All	13.3	12.0	3.2
Golden Retriever	F	16.0	16.0	-
	M	-	-	-
	All	16.0	16.0	-
German Shepherd	F	17.5	14.0	12.8
	M	13.6	11.0	10.2
	All	16.3	13.0	12.1
Hungarian Vizla	F	-	-	-
	M	15.0	15.0	-
	All	15.0	15.0	-
Labrador Retriever	F	15.2	13.0	7.3
	M	11.7	12.0	3.9
	All	14.3	12.5	6.7
Rottweiler	F	12.7	11.0	9.1
	M	11.3	9.0	6.4
	All	12.2	10.0	8.2
Rhodesian Ridgeback	F	10.1	10.5	3.1
	M	9.0	7.5	4.8
	All	9.6	10.0	3.8
All	F	16.2	13.0	11.6
	M	12.8	11.0	8.9
	All	15.1	12.0	10.9

HD=Hip dysplasia

Dogs aged between 12 and 24 months recorded a mean hip score of 15.3 (n=418; median 12.0) whereas those aged 25-48 months and more than 48 months had mean hip score of 14.7 (n=143; median 13.0) and 14.4 (n=30; median 13.0), respectively.

Moderate to severe HD (hip score >18) was diagnosed in 16.6% of dogs. On the other hand, mild HD (hip score 13-18) affected 32.7% of dogs, borderline HD (hip score 9-12) 37.7%, fair HD (hip score 7-8) 6.9%, and good HD (hip score 1-6) 6.1% of dogs (Tables-3 and 4).

**Table-3:** HD score grades of dogs with their frequencies over the years in a retrospective study on the canine HD scheme in Kenya (1998-2014).

HD score grade	Year																	Total (%)

	1998	1999	2000	2001	2002	2003	2004	2005	2006	2007	2008	2009	2010	2011	2012	2013	2014	
>18	1	2	2	7	4	2	5	3	5	9	6	11	7	12	10	4	8	98 (16.58)
13-18	3	8	6	17	9	3	4	19	15	19	6	12	14	14	16	14	14	193 (32.66)
9-12	-	3	6	8	3	2	11	23	21	22	12	28	14	15	26	3	26	223 (37.73)
7-8	2	-	-	-	2	1	4	5	3	2	1	5	3	5	2	3	3	41 (6.94)
4-6	1	-	-	1	-	-	-	6	3	4	5	3	3	3	3	-	3	35 (5.92)
1-3	-	-	-	-	-	-	-	-	-	-	-	1	-	-	-	-	-	1 (0.17)
Total	7	13	14	33	18	8	24	56	47	56	30	60	41	49	57	24	54	591

HD=Hip dysplasia

**Table-4:** HD score grades of dogs of different breeds in a retrospective study on the canine HD scheme in Kenya.

HD score grade	Breed									Total

	Boxer	Doberman	Great Dane	Golden Retriever	German Shepherd	Hungarian Vizla	Labrador Retriever	Rottweiler	Rhodesian Ridgeback	
>18	-	1	-	-	77		10	10	-	98
13-18	-	-	1	1	139	1	26	22	3	193
9-12	3	5	2	-	147	-	30	30	6	223
7-8	-	1	-	-	20	-	5	14	1	41
4-6	-	2	-	-	13	-	1	15	4	35
1-3	-	-	-	-	-	-	-	1	-	1
Total	3	9	3	1	396	1	72	92	14	591

HD=Hip dysplasia

## Discussion

The results of this study indicate that the number of dogs submitted by breeders and dog owners for HD screening has been increasing over the years. This trend could be attributed to the growing interest in dog breeding in response to the available market especially for security dogs and pets.

The three most predominant dog breeds were German Shepherds, Rottweilers, and Labrador Retrievers. This breed distribution was partly in agreement with previous study [10]. The German Shepherd breed, which is primarily a working dog, is widely known and used in the national security forces, private security companies, and homesteads. It is believed to be the most bred dog in the country. The German Shepherd breed mean score was higher than that of the total mean score in all dogs considered in this study suggesting that they are more predisposed to HD.

The prevalence and severity of canine HD were most likely related to the breed’s predisposition to the disease and the previous absence of a radiographic HD control program [11]. In many countries in the world, good results were achieved in the reduction of the HD frequency through controlled breeding of various dog breeds [12].

Regarding German Shepherd, the prevalence of HD was 19.44% (77/396). This percentage was lower than that reported by the OFA preliminary hip data (32.9%) and that reported by one study [10] which was 36.14%. Regarding Rottweiler, HD was recorded in 10.86% (10/99) of the screened dogs which was also lower than that reported by one study [10] which was 30.78% and OFA database (20.2%). In Labrador Retriever, HD was positive in 13.89% (10/72) of the cases which was also considered lower than that reported which was 27.23% [10] and slightly higher than that recorded by OFA database (11.7%). These differences could be attributed to the varied management practices in the different countries.

Most of the radiographs, which were brought for scoring, belonged to dogs that were between the ages of 12-24 months. This is a positive sign because the earlier poor hips are detected the better as they are eliminated from the breeding program. Moreover, therapeutic intervention may be done, if the dog is used for companionship.

The study also revealed that there were more females than males. The females also had a higher average hip score, which may be due to the fact that more females were kept in breeding kennels than males due to the whelping advantage. Moreover, one male was enough to service several females therefore bringing out the economic advantage. The tendency of females to have higher hip scores than males is probably an incidental finding simply due to the fact that more than 67% of the canine population considered in this study were actually females.

Two approaches of canine HD management has been described, which include conservative management and surgery [13]. Conservative management has been achieved by a combination of exercise restriction, weight control, analgesics, and physical therapies [14,15]. Surgery aims to prevent/limit the development of HD or reduce/eliminate pain through salvage. Surgical techniques that have been used with success include juvenile pubic symphysiodesis [13], pelvic osteotomy [16,17], pelvic ostectomy [18], denervation of hip joint capsule [13], shelf arthroplasty [19,20], intertrochanteric femoral osteotomy, excision arthroplasty, and total hip replacement [13]. However, the most effective way to reducing incidences of canine HD has been screening the defect and breeding against it [21,22].

Canine HD is a complex polygenic disease due to the small additive effect of many genes [23,24]. Environmental factors such as sex, age, and body weight have been reported to influence the expression and severity of the disease [25,26]. In addition, diet has been shown to have a significant effect on the development of HD in dogs predisposed to the disease and on the prevalence, severity, and clinical signs of osteoarthritis [10,13,27].

It is concluded that canine HD is present in Kenyan dogs and that the dog owners, breeders, and the Kennel Club have adopted and continue to implement the selection program of dog breeds free from HD on the basis of a devised hip scoring scheme. This helps to further decrease the frequency of HD.

## Authors’ Contributions

The data was collected by PK, SWM, and RMC. Statistical analysis was performed by GK, TOA, WEM, ANK, and JDM. Manuscript was drafted and revised by PK. All authors read and approved the final manuscript.
